# Statistical inference for time course RNA-Seq data using a negative binomial mixed-effect model

**DOI:** 10.1186/s12859-016-1180-9

**Published:** 2016-08-26

**Authors:** Xiaoxiao Sun, David Dalpiaz, Di Wu, Jun S. Liu, Wenxuan Zhong, Ping Ma

**Affiliations:** 1 Department of Statistics, University of Georgia, 101 Cedar Street, Athens, 30602 USA; 2 Department of Statistics, University of Illinois at Urbana-Champaign, 725 South Wright Street, Champaign, 61820 USA; 3 Department of Statistics, Harvard University, One Oxford Street, Cambridge, 02138 USA

**Keywords:** Differentially expressed gene, Gene set enrichment, Analysis of variance, Smoothing spline, Penalized likelihood

## Abstract

**Background:**

Accurate identification of differentially expressed (DE) genes in time course RNA-Seq data is crucial for understanding the dynamics of transcriptional regulatory network. However, most of the available methods treat gene expressions at different time points as replicates and test the significance of the mean expression difference between treatments or conditions irrespective of time. They thus fail to identify many DE genes with different profiles across time. In this article, we propose a negative binomial mixed-effect model (NBMM) to identify DE genes in time course RNA-Seq data. In the NBMM, mean gene expression is characterized by a fixed effect, and time dependency is described by random effects. The NBMM is very flexible and can be fitted to both unreplicated and replicated time course RNA-Seq data via a penalized likelihood method. By comparing gene expression profiles over time, we further classify the DE genes into two subtypes to enhance the understanding of expression dynamics. A significance test for detecting DE genes is derived using a Kullback-Leibler distance ratio. Additionally, a significance test for gene sets is developed using a gene set score.

**Results:**

Simulation analysis shows that the NBMM outperforms currently available methods for detecting DE genes and gene sets. Moreover, our real data analysis of fruit fly developmental time course RNA-Seq data demonstrates the NBMM identifies biologically relevant genes which are well justified by gene ontology analysis.

**Conclusions:**

The proposed method is powerful and efficient to detect biologically relevant DE genes and gene sets in time course RNA-Seq data.

**Electronic supplementary material:**

The online version of this article (doi:10.1186/s12859-016-1180-9) contains supplementary material, which is available to authorized users.

## Background

RNA-sequencing (RNA-Seq) technology has become a preferred choice for studying transcriptomes [1, 2]. Compared to microarray, RNA-Seq provides a single nucleotide level measurement of mRNA expression levels. It offers the chance to detect novel transcripts by obtaining tens of millions of short reads. When mapped to the genome or reference transcripts, RNA-Seq data are summarized by a number of read counts. The huge number of read counts enables researchers to quantify transcriptomes in ultra-high resolution [3, 4].

To study the dynamics of genome-wide mRNA expression levels during a biological process, e.g., development, researchers often conduct time course RNA-Seq experiments. As in static RNA-Seq experiments (RNA-Seq taken irrespective of time), identifying differentially expressed (DE) genes across different treatments or conditions is still a key task in time course RNA-Seq experiments. Inferring DE genes in time course RNA-Seq experiments has a number of interesting challenges. First, the DE genes in time course data are those with different gene expression profiles along the time across treatments or conditions. However, most of the available methods treat expressions of a gene at different time points as replicates and test the significance of the mean expression difference between treatments or conditions irrespective of time, e.g., edgeR [5] and DESeq [6]. They thus fail to identify many DE genes with different profiles across time. Second, some methods have been developed recently to identify the DE genes with different expression profiles over time. A recent work by Oh et al. [7] models time dependency using a hidden Markov model. Such a model requires the Markov property. In particular, the Markov property states that the conditional dependency of prior information from all time can be simplified to the conditional dependency of prior information of *k* time points (*k*th order Markov chain). It is still unclear whether such Markov property holds for general time course RNA-Seq data. Finally, both edgeR and DESeq use the total read counts of each gene and model the variation of the read counts across the replicates at gene level. When RNA-Seq experiments do not have replicates or the number of replicates is small, the statistical significance tests in edgeR and DESeq have small degrees of freedom and may result in a high false discovery rate (FDR).

**Fig. 1:**
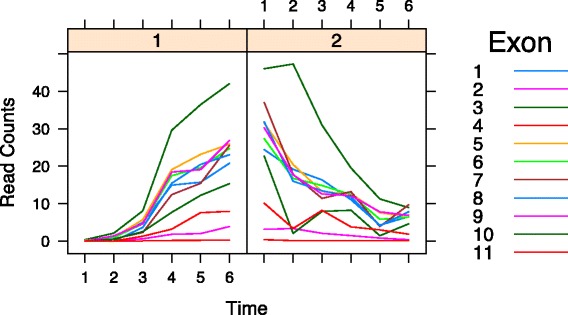
NPDE gene. Gene *ss* (FlyBase ID: FBgn0003513) was identified as non-parallel differentially expressed with *p* value=0.00. Different exons are represented by curves with varying colors. This gene participates in antennal development, antennal morphogenesis, and imaginal disc-derived leg segmentation. Read counts on the y-axis are the average counts (The total read counts on each exon divided by the length of exon). The left panel and right panel represent the early and late embryonic developmental stages respectively

**Fig. 2:**
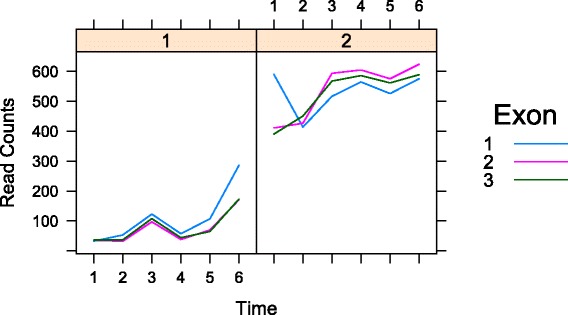
PDE gene. Gene *I* *d* *g* *f*2 (FlyBase ID: FBgn0020415) was identified as parallel differentially expressed with *p* value=0.00. Different exons are represented by curves with varying colors. This gene participates in imaginal disc development. Read counts on the y-axis are the average counts (The total read counts on each exon divided by the length of exon). The left panel and right panel represent the early and late embryonic developmental stages respectively

To surmount these challenges, we develop a novel statistical method to identify DE genes in this article. The input of our method is the read counts at the exon level for each gene at each time point. The read counts of genes at the exon level across different time points are modeled by a negative binomial mixed-effect model (NBMM). In this model, the mean gene expression profiles over time across treatments are modeled by a nonparametric bivariate function of time and treatments, while the time dependency is characterized by a parametric random effect. The nonparametric bivariate function has great flexibility in modeling different expression profiles over possibly non-equally spaced time points across treatments and conditions. The parametric random effects are used to define a variety of time dependency correlation structures. The model is fitted by a penalized likelihood method. In order to identify DE genes unique to time course experiments, we define two types of DE genes in time course RNA-Seq experiments: nonparallel differentially expressed (NPDE) genes with nonparallel expression profiles over time across treatments, see Fig. 1, and parallel differentially expressed (PDE) genes with parallel expression profiles over time across treatments, see Fig. 2. PDE genes are those consistently up-regulated or down-regulated over time across treatments, whereas NPDE genes are those that have significant expression profile changes over time across treatments. Compared with PDE genes, in many scientific investigations, NPDE genes are of primary interest. Focused study of the NPDE genes may provide more information on how the cell responds differently to different stimulus or treatments. Moreover, time course RNA-Seq experiments are commonly used in case-control studies and in clinical trials. In such experiments, mRNA samples are taken from a small number of subjects over time in the treatment group and from another small number of subjects in the control group. Because each group only consists of a small number of subjects, one subject with high baseline gene expression can cause a high average gene expression for the whole group. Thus, there are many PDE genes between treatments, but they are biologically irrelevant [8]. To distinguish the two types of DE genes, we decompose the nonparametric bivariate function in our model into the main effects of time and treatment separately, as well as their interaction through a functional ANOVA decomposition. The identification of DE genes is equivalent to testing significance of treatment-time interactions in the functional ANOVA decomposition. We fit this model to the exon level read counts data using penalized maximum likelihood. The tuning parameter is selected by cross-validation [9].

## Methods

### Nonparametric model and penalized likelihood method

**Fig. 3:**
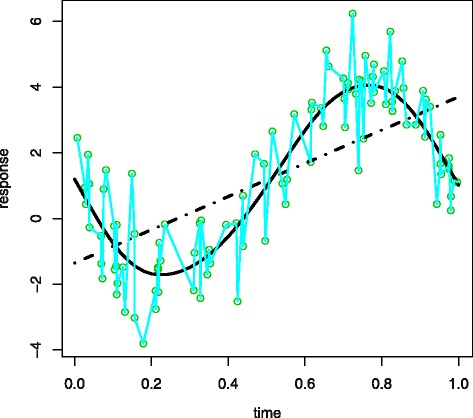
Curve fitting examples. The curve fitted using penalized likelihood is in the solid line and the linear fit is in dashed line, with the interpolation fit superimposed in faded line and the data in circles

We first provide a short review of nonparametric model and penalized likelihood method. Consider the nonparametric model for data points (*t*_*i*_,*y*_*i*_),

1 $$  y_{i} = \eta(t_{i}) + \epsilon_{i}, \quad i = 1,\cdots, T,  $$

where *η* is the mean function and random noise *ε*_*i*_ are independently Gaussian distributed. When *η* is assumed to be of form *η*(*t*_*i*_)=*t*_*i*_*β*, which is linear in *β*, one has a standard linear model. The disadvantage of linear model is illustrated by a toy example, where we generated 100 data points, faded circles in Fig. 3, from a nonlinear function. The linear model fit, the dashed straight line in Fig. 3, does not provide a good fit. Since linear model is too restrictive to model nonlinear function, we allow *η* to vary in a high-dimensional functional space, leading to diverse nonparametric estimators.

An approach to the estimation of *η* is via the minimization of negative log likelihood,

2 $$  \sum_{i=1}^{T}\left[y_{i} - \eta(t_{i})\right]^{2}.  $$

Without any constraint, the minimizer $\hat \eta $ in (2) simply interpolates the data and has no predicting power, see the faded line in Fig. 3. To avoid this problem, one uses penalized likelihood to get a smoothing estimator of *η* via minimization of

3 $$  \sum_{i=1}^{T}\left[y_{i} - \eta(t_{i})\right]^{2} + \lambda \int \left[\eta^{\prime\prime}(t)\right]^{2}dt,  $$

where *η*^′′^ is the second derivative and characterizes the smoothness of *η*,*λ* is a smoothing parameter, which controls the trade-off between the lack of fit of the nonparametric model and the roughness of *η*. To select the proper *λ*, researchers often use generalized cross validation [10]. An adequate fit by a proper selected *λ* is illustrated by the solid curve in Fig. 3.

### Negative binomial mixed-effect model

In time course RNA-Seq experiments, the short read counts cannot be adequately modeled by independent Gaussian distribution. We extend the aforementioned modeling strategy to develop a negative binomial mixed-effect model (NBMM) for modeling time course RNA-Seq data.

#### The model specification

Suppose the time course RNA-Seq experiments are conducted across *G* conditions/treatments. For each gene, the mapped read counts on exon *k* at time *t*_*i*_ in condition/treatment *g*, denoted by *Y*_*igk*_, are assumed to follow a negative binomial distribution (NegBin),

4 $$ Y_{igk} \sim \text{NegBin} (\nu, p(t_{i},g,k)),  $$

where the negative binomial distribution has the probability distribution,

5 $$ P(Y_{igk}=y)=\frac{\Gamma(\nu+y)}{y!\Gamma(\nu)}p(t_{i},g,k)^{\nu}(1-p(t_{i},g,k))^{y},  $$

where *ν* is a nuisance parameter, which is the number of reads that cannot be mapped to the reference genome, and 1−*p*(*t*_*i*_,*g, k*) is the probability that a read is mapped to exon *k* in condition *g* at time *t*_*i*_,*g*=1,⋯,*G, i*=1,⋯,*n*_*g*_,*k*=1,⋯,*K*. In this setting, *n*_*g*_ is the number of time points in the *g*th condition, and *K* is the number of exons. In most cases, we only have two treatments: case and control or mutant and wild type (*G*=2). To model the time trend and capture the time dependence, we use a nonparametric mixed-effect model with logit link ([11], p.199)

6 $$ {}\log\{p(t_{i},g,k)/(1-p(t_{i},g,k))\}=\log(\beta_{t_{i},g}) + \eta(t_{i},g)+{z}_{k}{b}_{k},  $$

where $\beta _{t_{i},g}$ is the effective library size, used in edgeR [12], of the *t*_*i*_th time point, mean expression *η* is assumed to be a smooth function of time *t* for each treatment *g*, *z*_*k*_ is the length of the *k*th exon, *b*_*k*_ represents the exon specific random effect to model the intra-exon variation with *b*_*k*_∼*N*(0,*σ*^2^), and the random effect variance *σ*^2^ is to be estimated from the data. The $\log (\beta _{t_{i},g})$ term provides a convenient device to normalize the reads to a common scale.

In model (6), the bivariate function *η* is decomposed as

7 $$ \eta(t,g) =\eta_{\emptyset}+\eta_{1}(t)+\eta_{2}(g)+\eta_{1,2}(t, g),  $$

where *η*_*∅*_ is the baseline expression irrespective of time and treatment, *η*_1_(*t*) is the time effect at time *t*, *η*_2_(*g*) is the treatment effect of the *g*th condition, and *η*_1,2_(*t, g*) is the interaction between time and treatment effects. The time and treatment effects are defined as the deviation from the baseline expression, and, therefore, ${\int _{0}^{T}}\eta _{1}(t)dt=0$ and $\sum _{g=1}^{G}\eta _{2}(g)=0$. Analogously, the time-treatment interaction is defined as ${\int _{0}^{T}} \eta _{1,2}(t,g)dt=0$ for all *g*, and $\sum _{g=1}^{G}\eta _{1,2}(t,g)=0$ for all *t*. This decomposition is referred to as the functional ANOVA decomposition [11, 13]. If the time-treatment interaction term *η*_1,2_(*t, g*) is significant, we have *η*(*t, g*_1_)−*η*(*t, g*_2_)=*η*_2_(*g*_1_)−*η*_2_(*g*_2_)+*η*_1,2_(*t, g*_1_)−*η*_1,2_(*t, g*_2_) for every *t*. In the right hand side, the first two terms are constants and the remaining terms vary with *t*. When the time-treatment interaction *η*_1,2_(*t, g*) is not significant in (7), the model reduces to

8 $$ \eta(t,g)=\eta_{\emptyset}+\eta_{1}(t)+\eta_{2}(g),  $$

which produces the parallel population mean time course profiles for different treatment conditions, i.e., *η*(*t, g*_1_)−*η*(*t, g*_2_)=*η*_2_(*g*_1_)−*η*_2_(*g*_2_) for each *t*, where the right hand side of the equation is a constant which does not vary with *t*. To distinguish the expression profiles, we define the genes with significant time-treatment interaction term in (7), i.e., *η*_1,2_(*t, g*)≠0, as non-parallel differentially expressed (NPDE) genes. If genes have a significant main effect in treatment *g* but no time-treatment interaction in (7), i.e., *η*_2_(*g*)≠0 and *η*_1,2_(*t, g*)=0, we define those as parallel differentially expressed (PDE) genes [8].

#### Estimation

By (5), one has a minus log likelihood

9 $$ \begin{aligned} &{}\sum_{k=1}^{K}\sum_{g=1}^{G}\sum_{i=1}^{n_{g}}\left\{\vphantom{\left(1+ e^{\log\{p(t_{i},g,k)/(1-p(t_{i},g,k))\}}\right)}(\nu+Y_{igk})\log\left(1+ e^{\log\{p(t_{i},g,k)/(1-p(t_{i},g,k))\}}\right)\right. \\ &\qquad\qquad-\!\!\left.\nu\log\left\{p(t_{i},g,k)/(1\,-\,p(t_{i},g,k))\right\}\!\!\vphantom{\left(1+ e^{\log\{p(t_{i},g,k)/(1-p(t_{i},g,k))\}}\right)}\right\}\!. \end{aligned}  $$

Substituting (6) into (9), we get the minus log likelihood of **Y** conditioning on random effects **b**, where $\phantom {\dot {i}\!}\mathbf {Y} =(Y_{111}, \cdots, Y_{n_{G},G,K})^{T}$, and **b**=(*b*_1_,⋯,*b*_*K*_)^*T*^. Therefore, the (Henderson) likelihood [14] of (**Y**,**b**) is

10 $$ \begin{aligned} &{}\log(f_{y|b}(\mathbf{Y} | \mathbf{b})f_{b}(\mathbf{b})) \\ &\propto\! \!\sum_{k=1}^{K}\!\sum_{g=1}^{G}\!\sum_{i=1}^{n_{g}}\!\left\{(\nu \,+\, Y_{igk})\log\left(\!1 \,+\,e^{\log(\beta_{t_{i}}) +\eta(t_{i},g)+z_{k}{b}_{k}}\!\right)\right. \\[-3pt] &-\left. \!\nu\left[\!\log(\beta_{t_{i}}) \,+\, \eta(t_{i},g)\,+\,{z}_{k}{b}_{k}\right]\!\vphantom{\left(1+ e^{\log\{p(t_{i},g,k)/(1-p(t_{i},g,k))\}}\right)}\right\} \,+\,\!\sum_{k=1}^{K}\!{b}_{k}^{2}\!/\!\sigma^{2}\!. \end{aligned}  $$

In (10), the *f*_*y*|*b*_ denotes the conditional distribution (negative binomial) of **Y** given **b**, and *f*_*b*_ denotes the distribution (normal) of **b**. In the end, we derive a penalized (Henderson) likelihood ([9], p.486) as

11 $$ \begin{aligned} {}\sum_{k=1}^{K}\!\sum_{g=1}^{G}\!\sum_{i=1}^{n_{g}}&\left\{(\nu \,+\, Y_{igk})\!\log\left(\!1 \,+\,e^{\log(\beta_{t_{i}}) + \eta(t_{i},g)+z_{k}{b}_{k}}\!\right) \right.\\[-5pt] &-\left.\!\nu\left[\!\log(\beta_{t_{i}}) \,+\, \eta(t_{i},\!g)\,+\,{z}_{k}{b}_{k}\right]\!\vphantom{\left(1+ e^{\log\{p(t_{i},g,k)/(1-p(t_{i},g,k))\}}\right)}\right\} \,+\,\!\!\sum_{k=1}^{K}\!{b}_{k}^{2}/\sigma^{2}\!\,+\,N\!\lambda{J}(\eta), \end{aligned}  $$

where $N=\sum _{k=1}^{K}\sum _{g=1}^{G}{n_{g}}$, the quadratic functional *J*(*η*) quantifies the smoothness of *η*, and the smoothing parameter *λ* controls the trade-off between the goodness-of-fit and the smoothness of *η*. The minimization of (11) is performed in a reproducing kernel Hilbert space $\mathcal {H} \subseteq \{\eta :J(\eta)<\infty \}$, in which *J*(*η*) is a square semi-norm [13]. For model (6) with functional ANOVA (7), we employ the following quadratic penalty, which produces a cubic spline estimate,

12 $$ {}J(\eta) \!= \!\theta_{1}^{-1} {\int_{0}^{T}}\left(d^{2}\eta_{1}/dt^{2}\right)^{2}\!dt + \theta_{1,2}^{-1}{\int_{0}^{T}}\!\sum_{g=1}^{G}\left(d^{2}\eta_{1,2}/dt^{2}\right)^{2}\!dt,  $$

where *θ*_1_ and *θ*_1,2_ are extra smoothing parameters that adjust the relative penalties on the roughness of different components. See detailed examples in Sect. 2.4 of [11]. For model (6) with functional ANOVA (8), we use penalty

13 $$ J(\eta) = {\int_{0}^{T}}\left(d^{2}\eta_{1}/dt^{2}\right)^{2}dt.  $$

To perform the penalized likelihood estimation of (11), we implement two nested iterative loops [9]. Fixing the smoothing parameter, the inner loop minimizes (11), and the outer loop estimates the smoothing parameters and variance of random effects via the minimization of certain cross-validation score, see [9] for details. For fixed smoothing parameter *λ*, (11) can be minimized through Newton iteration. Write

14 $$ l_{igk}(\zeta_{igk}) = (\nu + Y_{igk})\log(1 +e^{\zeta_{igk}}) -\nu \zeta_{igk},  $$

where $\zeta _{igk} = \log (\beta _{t_{i}}) + \eta (t_{i},g)+{z}_{k}{b}_{k}$. The quadratic approximation of *l*_*igk*_(*ζ*_*igk*_) at $\widetilde \zeta _{igk}$ is

15 $$ \begin{aligned} l_{igk}(\zeta_{igk})& \!\approx \!l_{igk}(\widetilde \zeta_{igk}) \,+\, \widetilde \mu_{igk}(\zeta_{igk}\,-\,\widetilde \zeta_{igk}) + \widetilde \omega_{igk}(\zeta_{igk}-\widetilde \zeta_{igk})^{2}/2 \\ & = \widetilde \omega_{igk}(\widetilde Y_{igk} - \zeta_{igk})^{2}/2 + E_{igk}, \end{aligned}  $$

where $\widetilde Y_{igk} = \widetilde \zeta _{igk} - \widetilde \mu _{igk}/\widetilde \omega _{igk} $ and *E*_*igk*_ is independent of *ζ*_*igk*_; $\widetilde \mu _{igk} = (\nu +Y_{igk})\widetilde p(t_{i}, g, k) - \nu $ and $\widetilde \omega _{igk} = \nu (1-\widetilde p(t_{i}, g, k))$. The Newton iteration can thus be performed via iterated weighted least squares,

16 $$ \begin{aligned} \sum_{k=1}^{K}\sum_{g=1}^{G}\sum_{i=1}^{n_{g}}\widetilde \omega_{igk}&(\widetilde Y_{igk}-\log(\beta_{t_{i}}) + \eta(t_{i},g)+{z}_{k}{b}_{k})^{2} \\&+\sum_{k=1}^{K}{b}_{k}^{2}/\sigma^{2}+N\lambda{J}(\eta). \end{aligned}  $$

Since *ν* is unknown, we estimate it from data. We apply the log operation to (5), and drop the terms that do not involve *ν* to get the individual objective function. Then the joint objective function is the sum of minus individual objective functions,

17 $$ \begin{aligned} \frac{1}{N}\sum_{k=1}^{K}\sum_{g=1}^{G}\sum_{i=1}^{n_{g}}\left\{\log(\Gamma(\nu))\,-\, \log\Gamma(\nu+Y_{igk})\,-\,\nu \log(p(t_{i},g,k))\right\}, \end{aligned}  $$

where *Γ* is the gamma function. Given (*Y*_*igk*_,*p*(*t*_*i*_,*g, k*)), one estimates *ν* via the minimization of (17). We iterate between the estimations of *η* and *ν* in (11) and (17) [11].

### Significance testing for individual gene

Once the model (6) is fitted to the exon level read counts data, we identify NPDE and PDE genes by testing the significance of the interaction and main effects in (7). To identify NPDE genes, we test the significance of the time-treatment interaction in (7), which is,

18 $$  H_{0}: \ \eta_{1,2}(t, g) =0; \ \ H_{1}: \ \eta_{1,2}(t, g) \neq 0.  $$

To derive the needed test statistic, we first define the Kullback-Leibler distance [11]

19 $${} \begin{aligned} &KL(\eta, \hat{\eta})\\&=\!\frac{1}{N}\sum_{k=1}^{K}\sum_{g=1}^{G}\sum_{i=1}^{n_{g}}\left\{\frac{\nu}{p(t_{i},g,k)} \log\frac{1-p(t_{i},g,k)}{1-\hat{p}(t_{i},g,k)} \,+\,\nu(\eta(t_{i},g)-\hat{\eta}(t_{i},g))\right\}. \end{aligned}  $$

Then, we use the following Kullback-Leibler distance ratio (KLR) [15] as our test statistic

20 $$  KLR=\frac{KL(\hat{\eta}_{F}, \hat{\eta}_{R})}{KL(\hat{\eta}_{F}, \eta_{C})},  $$

where $\hat {\eta }_{F}$ stands for a full model estimate given that *H*_1_ is true in the ANOVA decomposition (7), and $\hat {\eta }_{R}$ represents a reduced model estimate under the hypothesis that *H*_0_ is true in (7). Analogously, we define *η*_*C*_ as a constant function. For genes that are not considered as NPDE by the preceding test, we further investigate whether they are PDE or not. In model (6) with functional ANOVA (8), we are interested in testing

21 $$  H_{0}: \ \eta_{2}(g) =0; \ \ H_{1}: \ \eta_{2}(g) \neq 0.  $$

In testing for PDE genes, the full model estimate $\hat {\eta }_{F}$ does not include a time-treatment interaction, and $\hat {\eta }_{R}$ only has an overall mean and time effect in (8).

The *p* values for identifying NPDE and PDE genes are calculated through a permutation procedure. First, we compute a Kullback-Leibler distance ratio *KLR* for a gene. Second, the time labels for the gene are shuffled, and we recompute the statistic for the shuffled gene. We repeat the second step *B* times to obtain $KLR^{*}_{1}, \cdots KLR^{*}_{B}$. In the end, the *p* value for the gene is given by,

22 $$  \#\left\{KLR^{*}_{i} > KLR, i=1,\cdots,B\right\}/B,  $$

where *#*{·} represents the cardinality of the set, i.e., the number of permuted *KLR*^∗^s which is larger than the *KLR*.

### Gene set significance testing

In many studies, researchers are not only interested in identifying individual DE genes, but also in finding DE gene sets. A gene set may be defined by known biological information, for instance, a group of genes within the same biological pathway. Since genes within the same gene set are closely related, we increase statistical power of significance tests by borrowing information across genes. In addition, we obtain more robust results from gene sets than from individual genes. Subramanian et al. [16] proposed an approach named Gene Set Enrichment Analysis (GSEA), which tested the significance of pre-defined gene sets through a Kolmogorov-Smirnov like test. Efron and Tibshirani [17] proposed gene set analysis (GSA), which was shown to make a significant improvement over GSEA.

Following the ideas from GSEA and GSA, we test for significant NPDE gene sets via the following steps. Initially, pre-defined gene sets *S*_1_,*S*_2_, …, *S*_*P*_ are collected. Then, we compute the Kullback-Leibler distance ratio *KLR* based on (20) for all genes. For each gene set, *S*_*k*_, we calculate a gene set score, *R*_*k*_, defined as the average of the Kullback-Leibler distance ratios in (20),

23 $$  R_{k}=\sum_{i \in S_{k}}KLR_{i}/\#\{S_{k}\},  $$

where *#*{*S*_*k*_} is the number of genes in gene set *S*_*k*_. The gene set score *R*_*k*_ defines an enrichment test statistic, with a larger value of *R*_*k*_ suggesting a greater enrichment of NPDE genes. The PDE gene sets can be tested in the same way.

To test the significance of the gene set, a threshold is needed. The following permutation procedure is used to determine the threshold, and gene sets with values of *R*_*k*_ above the threshold are declared significant. In particular, we shuffle the time label for each gene and recompute the statistic for each permuted gene. We utilize formula (23) to calculate the permuted gene set scores $R^{*}_{1}, \cdots, R^{*}_{B}$, where *B* is permutation times. In the end, we calculate the *p* value of the *k*th gene set, given by,

24 $$  \#\left\{R^{*}_{i} > R_{k}, i=1,\cdots,B\right\}/B.  $$

## Results

### Simulation study

We evaluated the performance of the proposed method by carrying out extensive analysis on simulated datasets. Datasets were generated from both the NBMM model and an RNA-Seq simulator. All *p* values were adjusted by Benjamini and Hochberg (BH) method for multiple testing corrections [18].

#### Single gene simulation

We simulated exon level read counts according to Eqs. (4), (5) and (6). The effective library sizes of all time points were estimated by edgeR. We have three settings in this section. For each setting, *b*_*k*_∼*N*(0,1),*k*=1,2,3, accounts for variation of different exons, *z*_1_=0.1,*z*_2_=0.25 and *z*_3_=0.4 and *ν* is set to be 1000 for all those settings. Each exon was simulated with both single replicate and three replicates.

##### First setting: linear pattern.

**Fig. 4:**
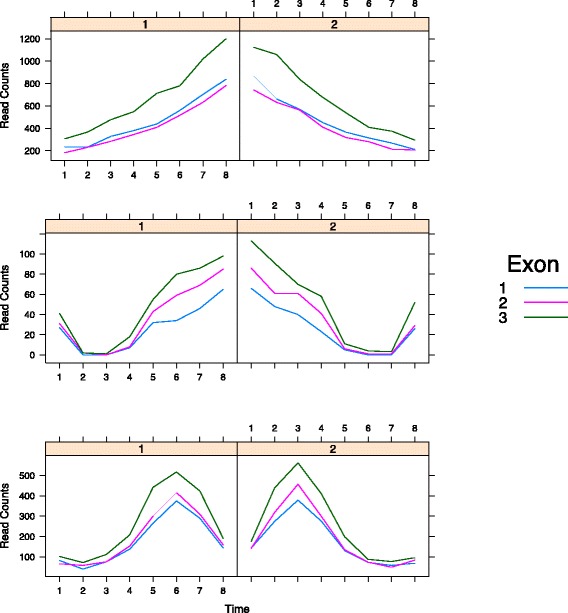
Simulated read counts. Simulated read counts generated from a negative binomial distribution. Samples of DE genes in the first, second and third setting are shown in the top, middle and bottom panel respectively. Different exons are represented by curves with varying colors

In the first setting, we generated exon level read counts of DE genes, see the top panel in the Fig. 4, using the following function,

25 $$  \eta(t_{i},g) = C((0.9-2t_{i})I_{[g=2]} + t_{i}),  $$

where *t*_*i*_=*i*/10,*i*=1,2⋯,8,*g*=1,2, and *C*=2 is a scale factor, *I*_[*g*=2]_ is an indicator function which equals one when *g*=2 and zero otherwise.

##### Second setting: exponential pattern

In the second setting, we simulated exon level read counts of DE genes, see the middle panel in the Fig. 4, using the following smooth function,

26 $$  \eta(t_{i},g) = \exp\left\{10^{4}F_{1}^{11}{F_{2}^{6}} + 10^{2}{F_{1}^{3}}{F_{2}^{9}}+ C_{g}\right \},  $$

where *F*_1_=(0.9−2*t*_*i*_)*I*_[*g*=2]_+*t*_*i*_,*F*_2_=0.1*I*_[*g*=2]_+*I*_[*g*=1]_+(1−2*I*_[*g*=1]_)*t*_*i*_, and *C*_1_=*C*_2_=1. The constants *C*_*g*_,*g*=1,2, define fixed reference expression levels for different conditions.

##### Third setting: cyclic pattern

In the third setting, exon level read counts of DE genes, see the bottom panel in Fig. 4, were generated using the following smooth function,

27 $$  \eta(t_{i},g) =\sin(2.5\pi((0.9-2t_{i})I_{[g=2]} + t_{i}) + 2.  $$

There were two scenarios in each setting. In the first scenario, we simulated time course exon level read counts of 50 genes. Half of the genes were DE genes generated by the above mean functions, and the remaining genes were generated as non-differentially expressed (NDE) genes by using the same mean function for different conditions. In the second scenario, 25 DE genes had the same profiles as those in the first scenario and 225 NDE genes were modeled as flat profiles. We compared the NBMM with three methods, maSigPro [19], DyNB [20] and edgeR. The former two methods are designed for time course data. Analysis followed the steps described in the R package documentation and unless stated otherwise default parameters were used.

Table 1 summarizes the performance of each method. The FDR was calculated as the number of false positives divided by the number of identified DE genes, and the False Non-Discovery Rate (FNR) as the number of false negatives divided by the number of genes which were not identified as DE genes. DyNB was only applied to the simulated data set of the first scenario in each setting due to its extensive computational cost, see Table 2. In the third setting, the DyNB failed to report the results for the data set with one replicate. In addition, edgeR was not recommended for single replicate data sets and, therefore, not used in each single replicate dataset [19].

**Table 1:** The FDR and FNR of all methods for detecting DE genes in simulation studies. If the method failed to report any significant genes, the FDR was NA and FNR was 0.50 for scenario 1 and 0.09 for scenario 2

			Setting 1		Setting 2		Setting 3	
			FDR	FNR	FDR	FNR	FDR	FNR
NBMM	Scenario 1	1 Rep	**0.00**	**0.00**	0.00	0.17	**0.00**	**0.14**
		3 Rep	**0.00**	**0.00**	**0.00**	**0.00**	**0.00**	**0.14**
	Scenario 2	1 Rep	0.07	0.00	0.00	0.02	**0.21**	**0.02**
		3 Rep	0.16	0.00	0.15	0.01	**0.09**	**0.02**
maSigPro	Scenario 1	1 Rep	0.11	0.00	**0.00**	**0.00**	NA	0.50
		3 Rep	0.00	0.07	0.00	0.04	NA	0.50
	Scenario 2	1 Rep	**0.00**	**0.00**	**0.00**	**0.00**	NA	0.09
		3 Rep	0.00	0.01	0.00	0.01	NA	0.09
DyNB	Scenario 1	1 Rep	NA	0.50	0.00	0.36	NA	0.50
		3 Rep	0.54	0.54	0.32	0.32	0.43	0.20
	Scenario 2	1 Rep	NA					
		3 Rep	NA					
edgeR	Scenario 1	Rep 1	NA					
		3 Rep	0.50	NA	0.50	NA	0.50	NA
	Scenario 2	1 Rep	NA					
		3 Rep	0.88	0.00	**0.00**	**0.00**	0.86	0.00

The best result in each scenario is shown in boldface

**Table 2:** The running CPU time (seconds) for all methods in simulation studies

		Setting 1	Setting 2	Setting 3
NBMM	1 Rep	7.133	6.182	7.261
	3 Rep	6.240	6.271	7.000
maSigPro	1 Rep	0.215	0.025	0.200
	3 Rep	0.235	0.091	0.236
DyNB	1 Rep	31944.470	NA	42513.210
	3 Rep	36228.200	36335.970	40412.250
edgeR	1 Rep	0.004	0.001	0.001
	3 Rep	0.001	0.001	0.001

The performance of edgeR, DyNB and maSigPro in terms of FDR and FNR was not as good as that of NBMM in the first scenario. This is expected since edgeR is not designed for time course data and the accuracy of detecting DE genes is affected by the estimated effective library size. When the NDE genes do not show flat profiles, the prediction performance of edgeR and maSigPro relying on TMM normalization [12] will be impaired. maSigPro had a better performance compared with NBMM method in the second scenario in linear and exponential settings. However, our method performed much better than other methods in more complicated patterns, such as a cyclic pattern. For this pattern, other methods either failed to detect any DE genes or identified almost all the genes as DE genes. In particular, in the first setting, the proposed NBMM method identified all DE genes. In the third setting, our approach identified about 88 % of DE genes with FDR 0.00 in the first scenario, whereas the maSigPro failed to detect any DE genes. In summary, as the pattern of the mean function moves away from linear to nonlinear, the advantage of the NBMM over other methods is getting more significant in detecting DE genes.

The NBMM took 7 s (CPU time) to process 50 genes with three replicates. Running CPU time for other settings are shown in Table 2. In summary, edgeR is not designed for time course RNA-Seq data, and, therefore, their performance is not as good as that of the NBMM and maSigPro in most settings. The maSigPro is applicable to time course RNA-Seq data and has a good performance in the roughly linear pattern. Its performance in the highly nonlinear pattern is not as good as the NBMM.

#### Simulation using RNA-Seq simulator

An RNA-Seq simulator, polyester [21], was applied to simulate RNA-Seq experiments. The simulator takes a set of annotated transcripts as input and produces files containing simulated RNA-Seq reads after simulating the steps of an RNA-Seq experiment. The reference genome used in the simulation was from *Drosophila melanogaster*. Tophat [22], samtools [23] and DEXSeq [24] were utilized to estimate the read counts data from the simulated fasta files. Analysis followed the steps described in the documentations and unless stated otherwise, default parameters were used.

We simulated the data of 7763 transcripts. By directly specifying the number of reads in each transcript, we simulated two expression patterns, linear expression pattern in (28) and nonlinear expression pattern in (29). In each pattern, 125 DE genes were created.

28 $$  v_{t_{i},g} = r((5-t_{i})I_{[g=2]} + t_{i}),  $$

where *r* is the reference expression level defined in (30) and *t*_*i*_=1+3(*i*−1)/7.

29 $$  v_{t_{i},g} = r(\sin(2.5\pi((0.9-2t_{i})I_{[g=2]} + t_{i}) + 2).  $$

The reference expression level is

30 $$  r = 20\iota/\upsilon,  $$

where *ι* is the length of transcript and *υ*=100 is the length of short reads. The expression values for NDE genes in all time points are defined in (30).

Removing genes with zero expression values over all time points, we came down with a data set including 4526 genes, among which 219 genes were DE genes.

We applied NBMM, maSigPro and edgeR to the dataset and results were summarized in Table 3. NBMM and maSigPro detected all DE genes with linear change pattern, however, NBMM identified 40 DE genes with nonlinear pattern whereas maSigPro found no genes with this pattern. As we can see in Table 3, the FDR and FNR of NBMM are lower than those of maSigPro. edgeR identified almost all the genes as DE genes and resulted in a higher FDR in Table 3.

**Table 3:** The FDR and FNR of all methods for detecting DE genes in simulation using polyester

	FDR	FNR
NBMM	**0.621**	**0.018**
maSigPro	0.737	0.028
edgeR	0.925	0.00

The best result in each scenario is shown in boldface

#### Gene sets simulation

**Fig. 5:**
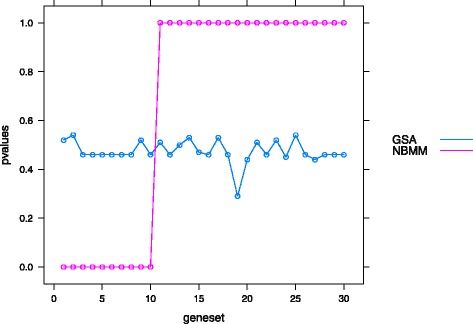
A comparison between result of NBMM and that of GSA. The *p* values of the proposed method are shown as pink cycles. The *p* values from GSA are shown as blue circles. The x-axis represents the gene set index, and the first 10 gene sets are the NPDE gene enriched gene sets

In this study, we simulated 30 gene sets, each with ten genes. All 100 genes in the first ten gene sets were NPDE genes generated by the first setting in (25). The rest of the gene sets were NDE genes with the same mean function for two conditions. We chose *ν*=1000,*C*=2 and calculated the gene set scores and *p* values for the simulated data. The R package GSA developed in [17] was used to detect DE genes enriched gene sets. In GSA package, we set *method*=“mean”, *minsize*=10, *resp*.*type*=“two class unpaired” and other parameters as default. The *p* values for all 30 gene sets calculated by NBMM and GSA are plotted in Fig. 5. The NBMM method detected all NPDE genes enriched gene sets, whereas the GSA method did not identify any significant gene sets.

### Real data analysis

Study of the development of *Drosophila melanogaster* (fruit fly) is important since this biological process shares many common features among different organisms. Graveley et al. [25] reported a time course RNA-Seq experiment of *Drosophila melanogaster* embryogenesis. The dataset included 12 embryonic samples collected at 2-hour intervals for 24 h. Each sample was collected at different stages of development. Sequencing was performed using the Illumina Genome Analyzer II platform. Reads of length 75 were uniquely aligned to the *Drosophila melanogaster* r5 genome using Bowtie [26].

Since in the first six time points, fruit flies were in the cleavage and gastrulation processes, whereas in the remaining six time points, they were in the process of differentiation [27], we divided the 12 time points into two developmental stages: early and late embryonic developmental stages. After data screening [5], the dataset used in our analysis consists of 1900 genes with different numbers of exons. Among these 1900 genes, 161 genes are related to embryo development (GO: 0009790) [28]. We aim to identify DE genes between the two developmental stages and find the significant pathways.

#### Single gene testing

The NBMM model was fitted gene-by-gene and the KLRs were calculated. The permutation procedure was used to obtain the *p* value for each individual gene. After multiple testing corrections, our method identified 192 NPDE genes and 751 PDE genes at a significance level of 0.05. We conducted functional annotation clustering for these genes using DAVID [29]. For NPDE genes, eight annotation clusters with enrichment scores above 2.0 were found. Seven of them are related to embryo development. For PDE genes, ten annotation clusters with enrichment scores above 2.0 were found. These clusters are associated with the regulation of RNA splicing, mitosis, and development related pathways.

**Fig. 6:**
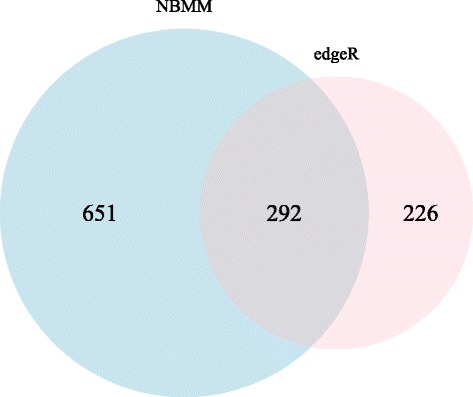
A comparison between the result of NBMM and that of edgeR. The Venn diagram between the sets of DE genes identified by NBMM and edgeR

Moreover, edgeR was applied to this dataset and 518 DE genes were found. There were 292 genes in common between the edgeR and proposed approach, see Fig. 6. Therefore, 651 DE genes were specifically found by NBMM and 226 DE genes were identified exclusively by edgeR. Among 161 genes in embryo development (GO: 0009790), 86 genes were identified by NBMM method, whereas edgeR detected 39 genes. For genes exclusively selected by edgeR, only two clusters with enrichment scores above 2.0 were found. These clusters are associated with certain catabolic processes. However, there were 11 clusters with enrichment scores above 2.0 for DE genes exclusively identified by the NBMM method. The biological processes associated with the clusters are the regulation of mRNA processing, mitosis, nuclear division, determination of anterior/posterior axis, embryo, and neuroblast differentiation, etc.

**Fig. 7:**
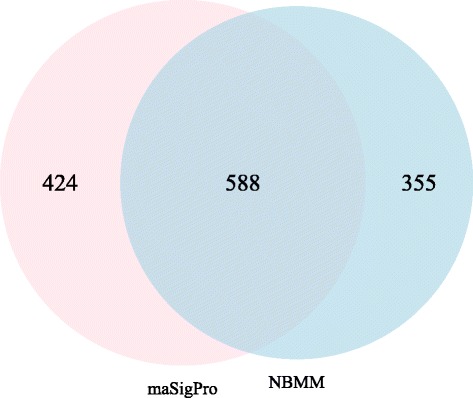
A comparison of the result of NBMM and that of maSigPro. The Venn diagram between the sets of DE genes identified by NBMM and maSigPro

In addition, we compared the NBMM with maSigPro, which detected 1012 DE genes. There were 588 genes in common between these two models, see Fig. 7. The NBMM specifically found 355 DE genes and 424 DE genes were identified exclusively by maSigPro. The annotation clustering was applied to these specifically identified DE genes. For genes exclusively selected by maSigPro, five clusters with enrichment scores above 2.0 were found. These clusters are associated with neuron projection morphogenesis, regulation of nuclear mRNA splicing and stem cell maintenance, etc. There were three clusters with enrichment scores above 2.0 for DE genes exclusively identified by the NBMM. The biological processes associated with the clusters are the mitosis, embryonic hindgut morphogenesis, gut development, etc. For the detailed functional annotation clustering, see the Additional files 1-6.

#### Gene sets testing

The pathway gene sets of the fruit fly were compiled using the Bioconductor package “org.Dm.eg.db”. The Entrez Gene identifier (version in Nov 2012) in each gene ontology term of org.Dm.egGO2ALLEGS was converted to official gene symbols using the org.Dm.egSYMBOL. We selected the gene sets with 15 to 30 genes and at least five of the 1900 genes were in the gene sets. We performed 100 permutations and chose pathways at the significance level of 0.05. Among 340 tested gene sets, 22 NPDE gene sets were selected by the NBMM, and 18 significant gene sets were selected by the GSA. Among 22 NPDE gene sets, eight gene sets are involved in the cell differentiation and cell development, see Table 4. The 18 significant gene sets detected by the GSA are the induction of apoptosis, chromosome localization, establishment of chromosome localization, cytoskeletal anchoring at plasma membrane, sarcomere organization, etc. These 18 gene sets are not associated with embryonic pathways. For the detailed information, see the Additional file 7. This shows that gene sets detected by the NBMM are more biologically relevant to development.

**Table 4:** The significant pathways identified by the NBMM gene set analysis of the fruit fly data

Pathway name	*p* value
Segment polarity determination	0.00
Salivary gland boundary specification	0.00
Glial cell differentiation	0.00
Glial cell development	0.00
Axon choice point recognition	0.00
Epithelial cell differentiation	0.00
Regulation of tube length, open tracheal system	0.00
Establishment of blood-brain barrier	0.00

## Discussion

Time course RNA-Seq data provide valuable insights into biological development and identifying biologically relevant DE genes is a key issue. We classify DE genes into two types: NPDE and PDE genes. Compared with PDE genes, NPDE genes are more likely to be biologically relevant. Therefore, focused study of the NPDE genes may provide more information on the underlying biological mechanisms. In this article, we proposed a statistical method, NBMM, for identifying DE genes in time course RNA-Seq experiments. Compared to other available methods, such as edgeR, the NBMM models time dependency and exon variation using a mixed-effect model. Moreover, the proposed NBMM method outperforms other approaches designed for time course RNA-Seq data in terms of DE genes detection accuracy, such as maSigPro and DyNB. The advantage of the NBMM over other competing methods is significant when they are applied to single replicate time course RNA-Seq data. Furthermore, gene sets significance test is shown to effectively detect DE gene sets.

The NBMM method is applied to gene expression data on a gene-by-gene basis. Thus, parallel computing can be employed for testing the significance of multiple genes simultaneously. We implemented a parallel computing option in our timeSeq package to speed up the computing process.

## Conclusions

In this paper, we developed a negative binomial mixed-effect model (NBMM) to detect the differentially expressed (DE) genes in time course RNA-Seq data. We showed that our approach outperforms other currently available methods in both synthetic and real data. The timeSeq, an open source software package, is freely available from CRAN.

## Additional files

Additional file 1 Functional Annotation Clustering for NPDE genes. (XLSX 12 kb)

Additional file 2 Functional Annotation Clustering for PDE genes. (XLSX 14 kb)

Additional file 3 Functional Annotation Clustering for genes specifically found by NBMM in comparison with edgeR. (XLSX 14 kb)

Additional file 4 Functional Annotation Clustering for genes specifically found by edgeR in comparison with NBMM. (XLSX 10 kb)

Additional file 5 Functional Annotation Clustering for genes specifically found by NBMM in comparison with maSigPro. (XLSX 10 kb)

Additional file 6 Functional Annotation Clustering for genes specifically found by maSigPro in comparison with NBMM. (XLSX 11 kb)

Additional file 7 Significant gene sets detected by the GSA. (XLSX 44 kb)
